# Counteracting Immunosuppression in the Tumor Microenvironment by Oncolytic Newcastle Disease Virus and Cellular Immunotherapy

**DOI:** 10.3390/ijms232113050

**Published:** 2022-10-27

**Authors:** Volker Schirrmacher, Stefaan van Gool, Wilfried Stuecker

**Affiliations:** Immune-Oncological Center Cologne (IOZK), D-50674 Cologne, Germany

**Keywords:** active-specific immunotherapy (ASI), adoptive T cell immunotherapy, bone marrow (BM), cancer vaccine, dendritic cell (DC), interferon-I, memory T cell (MTC), TAA, tumor dormancy, therapy resistance

## Abstract

An apparent paradox exists between the evidence for spontaneous systemic T cell- mediated anti-tumor immune responses in cancer patients, observed particularly in their bone marrow, and local tumor growth in the periphery. This phenomenon, known as “concomitant immunity” suggests that the local tumor and its tumor microenvironment (TME) prevent systemic antitumor immunity to become effective. Oncolytic Newcastle disease virus (NDV), an agent with inherent anti-neoplastic and immune stimulatory properties, is capable of breaking therapy resistance and immunosuppression. This review updates latest information about immunosuppression by the TME and discusses mechanisms of how oncolytic viruses, in particular NDV, and cellular immunotherapy can counteract the immunosuppressive effect of the TME. With regard to cellular immunotherapy, the review presents pre-clinical studies of post-operative active-specific immunotherapy and of adoptive T cell-mediated therapy in immunocompetent mice. Memory T cell (MTC) transfer in tumor challenged T cell-deficient nu/nu mice demonstrates longevity and functionality of these cells. Graft-versus-leukemia (GvL) studies in mice demonstrate complete remission of late-stage disease including metastases and cachexia. T cell based immunotherapy studies with human cells in human tumor xenotransplanted NOD/SCID mice demonstrate superiority of bone marrow-derived as compared to blood-derived MTCs. Results from clinical studies presented include vaccination studies using two different types of NDV-modified cancer vaccine and a pilot adoptive T-cell mediated therapy study using re-activated bone marrow-derived cancer-reactive MTCs. As an example for what can be expected from clinical immunotherapy against tumors with an immunosuppressive TME, results from vaccination studies are presented from the aggressive brain tumor glioblastoma multiforme. The last decades of basic research in virology, oncology and immunology can be considered as a success story. Based on discoveries of these research areas, translational research and clinical studies have changed the way of treatment of cancer by introducing and including immunotherapy.

## 1. Introduction

Immunotherapy is a change of paradigm in the treatment of cancer. It focusses the attention in the fight against cancer on the immune system of the cancer patient and tries to translate knowledge from basic immunological research into new strategies of treatment. Such translational research is constantly generating new advances and approaches.

This invited manuscript is a contribution to the Section “State-of-the-Art Biochemistry in Germany”. The corresponding author (VS) is a german biochemist and immunologist, co-author SvG is specialist in paediatric hemato-oncology and co-author WS is specialist in pharmaceutical biology, tumor immunology and translational oncology. The review is authentic with a focus on own achievements embedded in related work and latest findings in the field.

Oncolytic viruses (OVs) are interesting anti-cancer agents with high tumor selectivity. They replicate in and kill cancer cells without damaging healthy cells. This review focusses on the avian OV Newcastle disease virus (NDV) with its inherent anti-neoplastic and immune stimulatory properties. Avian NDV is the most extensively characterized member of the avulaviruses due to the high mortality rate and economic loss caused by virulent strains in the poultry industry. This enveloped paramyxovirus contains a non-segmented, negative-sense, single-stranded RNA genome encoding 6 structural propteins (N, P, L, M, HN, F) involved in the viral life cycle that is limited to the cytoplasm of the host cell. A special property of oncolytic NDV consists of its potential of breaking therapy resistance in human cancer cells. It is therefore particularly suited to counteract the immunosuppressive effects of the tumor microenvironment (TME).

T cell based cancer-specific immune reactivity represents the basis of the types of immunotherapy discussed in this review: post-operative active-specific immunotherapy with NDV-modified vaccines (see 4.11. and 4.15.) and adoptive cellular immunotherapy (see 2.3. to 2.5.). We therefore start with T cells. Chapter 2 presents examples of spontaneous anti-tumor T cell mediated immune responses, as evidenced in particular in the bone marrow (BM). It also provides examples of adoptive T cell mediated immunotherapy studies in which immune memory T cells infiltrate the TME and lead to tumor rejection. Chapter 3 addresses cellular details of the immunosuppressive TME before in chapter 4 the effect of NDV on the various cell types of the TME is being described. While intratumoral application of NDV directly influences the TME, post-operative active-specific immunotherapy with NDV-modified vaccines stimulates tumor-reactive T cells which indirectly affect the TME. The fifth and final chapter reports upon clinical studies of immunotherapy, using glioblastoma multiforme (GBM) with its immunosuppressive TME as an example.

For introduction to the topic, two up-to-date reviews may be helpful, one concerning oncolytic NDV [1], the other cellular immunotherapy [2].

## 2. Spontaneous Anti-Tumor T Cell Responses and Cellular Immunotherapy Studies

The formation of a solid tumor by a neoplastic cell requires a support ecosystem, i.e., an appropriate TME to allow growth and prevent immune attacks. In absence of a TME, such a transformed cell can principally initiate spontaneous host immune responses via the innate and the adaptive immune system.

This paragraph provides evidence for spontaneous immune T cell reactivity from a well-defined murine tumor model system. It then presents latest insights into spontaneous anti-tumor immune responses in human. Cellular immunotherapy studies involve murine and human immune T cells.

### 2.1. Evidence for Spontaneous Anti-Tumor T Cell Responses from a Mouse tumor Model

The highly aggressive murine ESb lymphoma, when transplanted into syngeneic mice subcutaneously (sc) or intraperitoneally (ip) grows, metastasizes and kills the host within 2–3 weeks. When transplanted into the ear pinna (ie), however, a site with a high density of dendritic cells (DCs), the cancer cells induce a strong immune response which prevents tumor growth and metastasis [3]. In these immune mice, induced ESb cancer-reactive CD8+ memory T cells (MTCs) control tumor dormancy in the bone marrow (BM) and establish long-term systemic immune resistance upon sc tumor cell challenge [3].

When ESb cells were transfected with the bacterial lacZ gene, it was possible to follow single tumor cells in tissues such as lymph nodes, spleen and BM of ESb-lacZ ie transplanted mice. LacZ, coding for the enzyme ß-galactosidase (Gal), was not only a marker to visualize individual tumor cells but it also served as a surrogate tumor-associated antigen (TAA) that induced major histocompatibility complex (MHC) class I-lacZ-peptide specific CD8 cytotoxic T lymphocytes (CTL) [4].

Research on tumor dormancy in the BM revealed that BM can function as a priming site for spontaneous T cell responses against blood-borne antigens, including TAAs [4]. This was a surprise because textbook immunology teaches that BM is a primary lymphoid organ involved in hemato- and lymphopoiesis while secondary lymphoid organs like lymph nodes and spleen are involved in initiating and facilitating immune responses. Due to tumor-induced angiogenesis, solid tumors become connected to the blood circulatory system so that tumor cells and TAAs can enter the blood. Blood-derived naïve T cells can home to BM sinus endothelium, transmigrate into the parenchyma and interact there with resident CD11c+ DCs. The latter are highly efficient in taking up exogenous blood-borne antigen and processing it via MHC class I and class II (MHC-I and MHC-II) pathways. Upon scanning of the DCs for expression of blood-borne antigens, the T cells with the corresponding T-cell receptor (TCR) form clusters with the antigen-presenting cells (APCs) in BM stroma, become activated, proliferate and differentiate into MTCs. Both, the activated T cells and the MTCs can transmigrate back into the BM sinuses and recirculate via the blood.

A novel tumor model was established for the study of long-term protective immunity and immune T cell memory [5]. Tumor-reactive immune cells against ESb-lacZ tumor cells were generated from a naïve T cell repertoire by a well established ie priming/ip restimulation protocol and transferred to tumor-inoculated T cell deficient nude (nu/nu) mice. The cell transfer prevented tumor outgrowth and resulted in the persistence of a high frequency of Gal-specific CD8+ T cells in the BM and spleen as demonstrated by tetramer staining of CD8 T cells specific for an immunodominant Gal epitope [5]. In contrast, immune cell transfer without tumor cell challenge did not result in detectable levels of Gal-specific CD8 MTCs.

Long-term immune memory and tumor protection could be maintained over four successive transfers of Gal-primed T cells between tumor-inoculated nu/nu recipients. The Gal-specific CD8+ MTCs from the first transfer could be activated and recruited into the peritoneal cavity by ip tumor cell challenge. From there they were harvested for a second adoptive transfer together with tumor cell challenge. About four weeks later the Gal-specific MTCs had returned into a resting state and were detected in the BM. This long-term experiment (>6 months) with four rounds of antigenic restimulation and adoptive immune cell transfer demonstrated longevity and functionality of the MTCs [6]. The results also suggested that the BM microenvironment has special features that are of importance for the maintenance of tumor dormancy and immunological T-cell memory. A low level of persisting Gal antigen appeared to favour the selection of Gal-specific MTCs over irrelevant MTCs in BM niches of CD8+ MTCs [6].

Acquisition of a BM phenotype by recirculating and tissue-resident MTCs has been described [7]. Redirection to the BM of gene-modified T cells was reported to improve T cell persistence and antitumor functions [8]. A dynamic kinetic view of human T cell memory concluded that homeostasis of circulating, proliferating and resting MTCs is controlled by different rheostats: tissue-exit and tissue entry signals for circulating MTCs, proliferation-inducing signals for proliferating MTCs, and availability of a survival niche for tissue-resident, resting MTCs [9].

Primary CD4 and CD8 T-cell responses generated in BM are autonomous and can occur in the absence of classical secondary lymphoid organs (lymph nodes and spleen) [4]. In spite of the absence of molecular adjuvants, the BM T cell responses to blood-borne surrogate TAAs were not tolerogenic and resulted in the generation of CTLs. Primary T-cell responses in BM were also discovered in mice reconstituted with transgenic T cells from OT-I or OT-II mice specific for ovalbumin (OVA) [4]. The BM microenvironment, upon entry of antigens in absence of adjuvants, also facilitates DC (APC):CD4 T cell interactions and maintenance of CD4 memory [10,11]. Why the BM microenvironment does not require adjuvants to initiate primary T cell responses against blood-borne antigens is not yet clear but it is possible that naïve T cells in this environment are in a higher state of activation. An important role for BM as a secondary lymphoid organ was confirmed later by demonstrating high frequencies of Wilms tumor antigen 1 (WT1)-specific CD8+ T cells in BM from tumor-bearing patients [12].

BM represents an excellent site for long-term maintenance of memory CD4+ and CD8+ T cells due to special niches providing survival cytokines such as IL-7 and IL-15 [11,13]. The link between MTCs and stromal cells in survival niches is very robust and can provide efficient memory over a lifetime in tissues such as the BM [14].

Mutation-derived tumor neoantigens play an important role in generating spontaneous anti-tumor immune responses. In recent years molecular pathways have been identified which influence T cell immunoreactivity to tumor neoantigens and cancer immuno-editing. For example, durable CD8+ neoantigen-specific T cell immunity was discovered to be controlled through mRNA m^6^A and the YTHDF1 m^6^A binding protein in DCs involved in cross-priming [15]. Type I interferon (IFN-I) was found to activate APC function in DCs through IFN-stimulated genes (ISG+DCs). Unlike cross-presenting DC1, ISG+DCs acquire and present intact tumor-derived peptide-MHC (pMHC) complexes [16]. In addition to MHC-I neoantigens recognized by CD8+ T cells, MHC-II neoantigens recognized by CD4+ T cells have been identified [17]. These have a key function in shaping tumor immunity and response to immunotherapy.

### 2.2. Spontaneous Anti-Tumor Immune Responses in Cancer Patients

Spontaneous immune responses to TAAs or tumor neoantigens have been described not only in animal model systems but also in cancer patients. Immune cells can be isolated (i) from tumor samples as tumor-infiltrating lymphocytes (TILs), (ii) from peripheral blood derived mononuclear cells (PBMCs) or (iii) from BM derived mononulear cells.

Spontaneous anti-tumor immune responses in cancer patients could be analyzed from BM aspirate samples. BM samples from 39 primary operated breast cancer patients and 11 healthy females were analyzed for the presence and frequencies of spontaneously induced MTCs with peptide-HLA-A2-restricted reactivity against 10 breast tumor associated TAAs and 3 normal breast tissue-associated antigens in short-term IFN-γ enzyme-linked immunospot (ELISPOT) essays. 67% of the patients recognized TAAs with a mean frequency of 144 TAA reactive cells per 10^6^ T cells. Strong differences of reactivity were noticed between TAAs, ranging from 100% recognition of prostate-specific antigen (p141-149) to only 25% recognition of MUC1 (p12-20) or Her-2/neu (p369-377). Reactivity to normal breast tissue-associated antigens was low [18]. The study revealed the shaping of a polyvalent and highly individual BM T-cell repertoire in cancer patients [19]. 

Enrichment of MTCs and other profound immunological changes in the BM was reported from untreated breast cancer patients [20]. The proportion of MTCs among CD4+ and CD8+ T cells was much higher in BM from cancer patients than in BM from healthy donors. The extent of MTC increase was related to the size of the primary tumor. Patients with disseminated tumor cells in their BM had more memory CD4+ T cells and more CD56+CD8+ cells than patients with tumor cell-negative BM [20].

BM samples and peripheral blood from 41 pancreatic cancer patients were characterized for location, frequencies and functional potential of spontaneously induced MTCs specific for individual or common TAAs. Pancreatic cancer is highly malignant and dominated by Th2 cytokines in patients` sera suggesting systemic tumor-induced immunosuppression. Surprisingly, high numbers of tumor-reactive T cells were found in all BM samples and in 50% of blood samples. These cells secreted the Th1 cytokine IFN-γ upon stimulation with TAAs [21].

Detailed studies of cognate interactions between MTCs and APCs from the BM of cancer patients revealed bidirectional cell stimulation, survival and antitumor activity in vivo [19]. For example, IFN-α which can be induced in DCs by T cells, has a reciprocal effect on T cells by inducing the expression of IL-12 receptor ß, enabling the T cells to respond to IL-12 and to differentiate into Th1 cells. Other relevant cytokines in this cognate interaction between DCs and CD4+ and CD8+ T cells are IL-2, IFN-γ and TNF-α [22].

### 2.3. Therapy of Human Tumors in NOD/SCID Mice with Patient-Derived Reactivated MTCs from BM

Freshly isolated T cells from BM of breast and pancreatic cancer patients recognized autologous tumor cells and rejected them in a xenotransplant model demonstrating their functional and therapeutic potential [23]. In short-term culture with autologous DCs pre-pulsed with tumor lysates, patient`s MTCs from BM (but not from PBMC) could be specifically reactivated to IFN-γ producing and cytotoxic effector cells [23].

A single ip transfer of such restimulated BM T cells into NOD/SCID mice caused regression of autologous tumor xenotransplants. This immune response was associated with infiltration by human T cells and tumor cell apoptosis and necrosis. This demonstrated therapeutic efficiency in vivo of ex vivo re-activated BM-derived cancer-reactive MTCs from cancer patients. Transfer of BM derived CD45RA(-) MTCs but not CD45RA(+) naïve T cells infiltrated autologous tumor but not autologous skin tissue. The TILs had a central or effector memory phenotype and produced perforin. Many of them expressed P-selectin glycoprotein ligand 1 and were found around P-selectin (+) tumor endothelium. Tumor infiltration included cluster formation in tumor tissue by MTCs with co-transferred DCs. Depletion of DCs from restimulation cultures before transfer to NOD/SCID mice reduced therapeutic efficiency suggesting an important contribution of APC restimulation in tumor tissue [24].

These studies demonstrated selective homing of human MTCs to human tumors in xenotransplanted mice and suggested that tumor rejection is based on the recognition of TAAs on tumor cells and DCs by autologous specifically activated central and effector MTCs [23,24].

### 2.4. Therapeutic Potential of Cancer-Reactive MTCs from BM in Cancer Patients

A review from 2015 described the spontaneous induction of cancer-reactive MTCs from BM, their maintenance by the BM microenvironment and their therapeutic potential [25]. A pilot clinical study investigated adoptive immunotherapy of advanced metastasized breast cancer with BM-derived cancer-reactive MTCs [26]. The BM MTCs apparently had an extensive expansion capacity in the patients [25]. Immunological responder patients showed a significantly longer overall survival (OS) than nonresponders (median survival 58.6 vs. 13.6 months; *p* = 0.009) [27].

### 2.5. Cellular Immunotherapy Counteracting Advanced Metastasized Cancer

Effective immune rejection of advanced metastasized cancer was demonstrated in a graft-versus-leukemia (GvL) animal model of already cachectic mice [28]. In situ activated tumor-immune T cells, induced in allogeneic, tumor-resistant, MHC identical but superantigen different donor mice could transfer strong GvL effects accompanied by only mild graft-versus-host (GvH) reactivity. A single systemic immune cell transfer into 5 Gy irradiated cachectic DBA/2 mice bearing up to 4 week established syngeneic tumors and macrometastases led to massive infiltration of tumor tissue by CD4+ and CD8+ donor T lymphocytes. Primary tumors of 1.5 cm diameter were encapsulated and rejected from the skin and liver metastases were eradicated. For the first time such adoptive cellular immunotherapy was followed in individual live animals by ^31^P-NMR spectroscopy of primary tumors. This allowed to evaluate changes in tumor tissue pH. An approximately 25,000 fold excess of metastatic tumor cells could be rejected as revealed quantitatively by FACScan analysis of lacZ gene transfected tumor cells [28].

Lessons from such GvL studies in animals about complete remission of cancer in late-stage disease by radiation and transfer of allogeneic MHC-matched immune T cells, in particular of MTCs from the BM [29], were:

(i) reversion of tumor tissue pH from acid to neutral after 3–4 days as a first sign of the immunotherapeutic effect,
(ii) donor CD4 T cell infiltration in the tumors 6 days after cell transfer,
(iii) formation of a broad capsule of fibrous tissue between the tumor area and the skin,
(iv) tumor rejection and long-term survival,
(v) wound healing and scar tissue formation at sites of primary tumor rejection (skin) and at sites of metastases (liver and kidney),
(vi) reconstitution of normal fur at the site of rejected primary tumor,
(vii) cellular interactions: donor CD4+ and CD8+ immune T-T cell interactions, donor T cell-host macrophage interactions around liver metastases, vß6 donor T cells recognizing a tumor-associated viral superantigen (vSAG-7) interacting with tumor cells and APCs [29],
(viii) reversibility of a state of cachexia,
(ix) disproval of the hypothesis that a tumor is a never healing wound [30].

Table 1 lists the most important aspects presented in this chapter.

## 3. The Tumor Microenvironment

The TME consists of ECM and stromal cells, such as immune cells, mesenchymal cells (MSCs), cancer-associated fibroblasts (CAFs) and vascular endothelial cells. The ECM contains ECM proteins, type IV collagen, galectin-1, proteoglycans and glycoproteins. Type IV collagen is the major component of the basement membrane that separates epithelium and epithelium-derived tumors (carcinomas) from stroma. The key enzymes and inhibitors regulating ECM turnover are matrix metalloproteinases (MMPs) and tissue inhibitors of MMPs (TIMPs). Unlike cancer cells which transform through a series of genetic alterations, stromal cells are mostly genetically intact. However, stromal cells can become corrupted by malignant cells which try to create a microenvironment permissive for tumor growth and cancer progression [31].

Key findings which have defined the TME have been reviewed [31]. Among the nine key findings are the “seed and soil” hypothesis by Paget from 1889 and the discovery of tumor angiogenesis by Folkman and colleagues in 1971 [32].

We provide a few examples to demonstrate interactions of tumor cells with their TME. Such interactions serve to advance tumor cell invasive growth, to suppress or evade attacks by the immune system and to establish local or distant metastases. An understanding of cellular and molecular pathways that lead to an immunosuppressive TME is a prerequisite for targeted therapies against the TME.

### 3.1. BM TME

Solid tumors such as breast, prostate, and lung cancers frequently spread to bone, causing severe pain, disability and cancer-related deaths [33]. Tumor growth factor ß (TGF-ß), bone morphogenic protein (BMP) and Wint (Wnt) signaling pathways are key mediators of paracrine signaling between bone stromal cells and tumor cells. Bone metastases from solid tumors change the complex BM microenvironment [34]. Novel translational approaches targeting the BM microenvironment have recently been presented [34]. Clinical trials targeting bone metastasis pathways use (i) monoclonal antibodies (mAbs) against TGF-ß, CCL2 or CXCL8 chemokines or (ii) small molecule inhibitors (SMIs) as antagonists of the chemokine receptor CXCR4 or as inhibitors of MMPs involved in matrix remodeling [34]. Other emerging therapeutic targets are CXCR1, RANKL, PTHrP, VEGF, and LOX [34].

### 3.2. CAFs in the TME

Recent reviews highlight the complexity of CAF biology including CAF heterogeneity, functionality in drug resistance, TGF-ß mediated immune evasion, contribution to a progressively fibrotic tumor stroma, the involved signaling pathways and the participating genes [35,36,37].

Infiltration of the TME by modified stromal cells initiates remodeling of the tumor ECM through increased secretion of fibronectin and collagen and expression of ECM receptors, ultimately generating a modified fibrotic desmoplastic TME. CAF-assisted contractility of this tumor matrix induces signaling pathways (e.g., NF-kB, JAK/STAT3), in conjunction with the direct interaction of plasminogen activator inhibitor-1 (PAI-1) with respective cell surface receptors (LRP1, uPAR) on inflammatory and cancer cells. The clinical outcomes are multidrug resistance, cancer stem cell self-renewal, poor disease outcome and shorter disease-free survival [38].

TGF-ß modulates ovarian cancer invasion by upregulating CAF-derived versican (VCAN) in the TME [39]. VCAN expression by CAFs is regulated through TGF-ß receptor type II and SMAD signaling. Upregulated VCAN promotes the motility and invasion of ovarian cancer cells by activating NF-kB signaling pathway and by upregulating expression of CD44, MMP9, and a hyaluran-mediated motility receptor [39].

### 3.3. TME “Cold”, Barriers to T Cell Infiltration, Tumor Cell Defense Mechanisms against CTL Attack

T cell infiltration of tumors is affected by barriers such as the dense fibrogenic ECM and aberrant tumor vasculature. Tumor-infiltrating T cells, TILs, are also affected by immunosuppressive cytokines (e.g., IL-4, IL-10), growth factors (e.g., TGF-ß) [39], enzymes (e.g., indoleamine 2,3-dioxygenase (IDO)) and by immunosuppressive cells. The non-T cell-inflamed TME has been termed as “cold” [40].

CTLs eliminate tumor cells via a combination of killing modes [41]. Lethal hits at the lytic synapse between a CTL and a tumor cell lead within seconds to sustained Ca^2+^ release, damaged membrane and caspase 3 activity. Against this activity tumor cells can induce ultra-rapid defense mechanisms based on the synaptic lysosomal/late endosomal (LLE) membrane repair pathway [41]. To date, four modalities have been implicated in target cell death upon CTL attack: intrinsic apoptosis, extrinsic apoptosis, pyroptosis and ferroptosis. In addition to the ultra-rapid defense mechanisms, tumor cells are able to establish slower defense mechanisms (e.g., removing damaged organelles) and constitutive defense mechanisms (e.g., upregulation of inhibitors of apoptosis proteins) [41].

### 3.4. MDSCs and Immunosuppression

During tumor progression, immunosuppression is mediated, among others, by myeloid-derived suppressor cells (MDSCs) [42,43]. BM-derived immature myeloid cells (IMC) differentiate under steady-state conditions, into granulocytes, macrophages and DCs. Under chronic inflammatory conditions, typical for tumor progression, this differentiation is impaired, leading to accumulation of IMCs. Immunosuppressive pathways by MDSCs inhibit T cell functions through the expression of various cytokines and immune regulatory molecules, inhibition of lymphocyte homing, stimulation of other immunosuppressive cells, depletion of metabolites critical for T cell function, expression of ectoenzymes regulating adenosine metabolism and by the production of reactive oxygen or nitrogen species [42]. There are two major groups of MDSCs, namely granulocyte/polymorphonuclear MDSCs (PMN-MDSCs) and monocytic MDSCs (M-MDSCs). Major developmental factors for PMN-MDSCs are high levels of granulocyte-macrophage-colony-stimulating factor (GM-CSF), vascular endothelial growth factor (VEGF), IL-6, Il-1ß, adenosine and hypoxia-inducible factor α (HIF1α). Major developmental factors for M-MDSCs are high levels of macrophage-colony-stimulating factor (M-CSF), VEGF, adenosine and HIF1α [43].

### 3.5. MDSC, CAF and Neutrophil Involvement in the Pre-Metastatic Niche

MDSCs, CAFs and neutrophils contribute to the formation of the pre-metastatic niche by “priming” it [44]. Neutrophils or PMN-MDSCs are recruited to the pre-metastatic niches mostly through the chemokine receptors CXCR2 and CXCR4. Neutrophil extracellular traps (NETs) are extracellular structures released by neutrophils in response to stimuli. They are composed of cytosolic and granule proteins as well as DNA. In pancreatic cancer, NETs were reported to be triggered by tissue inhibitor of metalloproteinase 1 (TIMP1) [45]. Preparation of pre-metastatic niches includes matrix remodeling, immunosuppression, NETs, reactive oxygen species (ROS) production, inflammation and tumor cell recruitment. PMN-MDSCs also escort tumor cells into the circulation. This leads to increased metastatic potential, to inhibition of NK cells and to increased extravasation. NETs finally trap tumor cells into the microvasculature [44].

It is clear from this information that a TME is relevant not only for a primary tumor but also for secondary or tertiary sites, i.e., metastases. The “seed and soil” hypothesis by Paget already stated that metastases need a proper “soil” for being able to grow.

### 3.6. M2 TAMs

In the TME tumor-associated macrophages (TAMs) are abundant within stromal cells. TAMs are classified into two major subsets, M1-like and M2-like TAMs [46]. M1-like TAMs are activated by IFN-γ, TLRs, lipopolysaccharide and GM-CSF. M1-like TAMs strengthen a T-helper 1 response and secrete pro-inflammatory cytokines. M2-like TAMs are activated by IL-4 and IL-13. They enhance a T-helper 2 response and participate in the regression of inflammation and wound healing by secreting anti-inflammatory factors such as IL-10 and TGF-ß. M1-like TAMs secreting pro-inflammatory cytokines such as TNF-α, IL-1, IL-6, IL-12 and IL-23 have anticancer effects, while M2-like TAMs contribute to tumor progression [46].

Tumor-derived exosomal non-coding (nc) RNAs were found to induce M2 macrophage polarization through signaling pathway activation, signal transduction, and transcriptional and post-transcriptional regulation. Exosomes from TAMs also play a role in intercellular communication. TAM-derived exosomal ncRNAs promote tumor proliferation, angiogenesis, immunosuppression, chemoresistance and metastasis [47].

Signaling through signal transducer and activator of transcription 3 (STAT3) and NFkB was reported to mediate crosstalk between M2 TAMs and malignant cells. Disruption of such signals caused a switch from M2 phenotype (CD163) to M1 phenotype (CD68) associated with reduced levels of IL-10, TGF-ß and CCL22 [48].

### 3.7. Tolerogenic DCs

DCs play an important role in the TME [49]. Their absence or paucity characterizes “cold” TMEs such as those from pancreatic cancer [50]. The DCs may be tolerogenic or polarize the T cell response towards Th17. Factors regulating type 1 conventional DC (cDC1) function in the TME have been described [50]. cDC1 production of IL-12 can be directly inhibited by IL-10 released by macrophages or other immunosuppressive cells. Tumor-derived factors such as VEGF inhibit the maturation of cDC1 [51].

Tumors are frequently infiltrated by type 2 conventional DC (cDC2). Upon migration to the lymph node, these DCs are able to initiate CD4+ T cell responses if they are not restrained by T regulatory cells (Treg) [51,52].

Tolerogenic CD103+ DCs produce the enzyme IDO and secrete the cytokines IL-4 and IL-10 [53]. The acquisition of an immunosuppressive DC phenotype is tightly regulated by epigenetics [54].

### 3.8. Anergic T Cells and Treg Cells

A balance of positive and negative signals tunes the immune reactivity of T lymphocytes [55,56]. To avoid immune-mediated tissue damage and autoimmunity, the signals generated by immunoreceptors must be tightly controlled by negative signals [55]. The best studied T cell activating receptors are the TCR and CD28 and the best studied T cell inhibitory receptors are PD-1 and CTLA-4. Activating receptors signal via tyrosine kinases (Tyr kinases) (e.g., ZAP70 and LCK) to produce diacylglycerol (DAG) via PLCy1 and via phospho-inositol (PI) kinases such as PI3Kα. Conversely, inhibitory receptors signal via Tyr phosphatases (e.g., SHP-2) to metabolize DAG and via PI phosphatases (e.g., SHIP-1). The joined inhibitory action of PD-1 on the PI3K/AKT and the MAPK pathway results in transcriptional modulation of cell cycle progression [55,56].

Anergic T cells from the TME are characterized by low expression of the TCR, of perforin and of Fas-L [51]. TILs can either respond to anti-PD-1/PD-L1 immune checkpoint inhibition (ICI) or they can be resistant. Resistance mechanisms of TILs from solid tumors to ICI have been described [55]. They have to do with negative receptor signaling in T cells [56].

Rho G, a member of the Rac family of small GTPases, induces T cell anergy by promoting the activities of transcription factors, including nuclear factor of activated T cell (NFAT)/AP-1 [57].

Accumulation of Tregs in the TME has been correlated with poor prognosis in many solid tumors [58]. A recent study reveals that obstruction of antitumor immunity by Tregs is due to promotion of T cell dysfunction and to restricting clonal diversity in CD8+ TILs [58].

### 3.9. Dysfunctional NK Cells

Low numbers of dysfunctional NK cells are often observed in many advanced solid human cancers [59]. Potential mechanisms that influence suboptimal mature NK cell recruitment and function in the TME have been discussed [59]. The expression of major activating NK receptors, the NK cytolytic activity and the cytokine production were inhibited upon co-culture with PMN-MDSCs through cell-to-cell contact, soluble factors and exosomes [60].

Immunosuppressive genes in NK cells code for the IL-15 signaling inhibitor CIS (Cish) and for the immunosuppressive TGF-ß (Tgfbr2) [61].

### 3.10. Tumor-Derived Factors

Tumor-derived factors modulate the TME. They include growth factors (e.g., TGF-ß), immune inhibitory ligands (e.g., PD-L1), prostaglandins and lactic acid as by-product from tumor metabolism. A variety of cytokines, chemokines and growth factors are produced in the TME by different cells representing a complex ecosystem and network of cell interaction, regulation of differentiation, activation, function and survival or death of various cell types [62].

### 3.11. Metabolic Barrier, T Cells, Hypoxia and Tumor Dormancy

T cells encounter a hostile metabolic environment in tumors [63]. TILs isolated from clear cell renal carcinoma patients showed decreased glucose uptake as well as small, fragmented mitochondria with elevated ROS [63]. T cells primed in nutrient-rich lymphoid tissues enter tumors where cancer cell metabolism and poor vascular exchange lead to competition for resources. One hostile aspect of the TME is hypoxia created by the high metabolic rate of tumor cells in conjunction with inadequate vasculature. Under low oxygen states, the transcription factor HIF is free from its negative regulator von Hippel-Lindau (VHL) to upregulate its target genes [64].

T cells undergo metabolic re-programming in different stages of their life. 1. Naïve T cells take up glucose via the receptor Glut1. This fuels via pyruvate oxidative phosphorylation (OXPHOS) the tricarbonicacid (TCA) cycle. 2. Upon cognate antigen encounter on APCs, T cells become activated and rapidly take up more glucose and additionally glutamine to fuel their bioenergetic needs. Activated T cells perform aerobic glycolysis, which shunts products of glycolysis to biosynthetic processes necessary for proliferation and effector function. Like tumor cells, activated T cells produce lactate as a byproduct. 3. Once the antigen is cleared, T cells can form long-lived MTCs. In memory cells AMP-activated protein kinase (AMPK) signaling stimulates fatty acid oxidation. The fatty acids are synthesized upon uptake of glycerol. MTCs also increase their mitochondrial mass and spare respiratory capacity to prepare for future encounter with cognate antigen. 4. T cells can become exhausted if they fail to clear antigens such as during chronic infections or cancer. TILs isolated from tumors display elevated levels of PD-1. This decreases PI3K/AKT/mammalian target of rapamycin (mTOR) signaling and glycolysis. Exhausted TILs often have dysfunctional mitochondria and decreased mitochondrial mass [63].

A recent study revealed that TCR-induced upregulation of Myc-dependent glycolytic metabolism in murine CD8+ T cells is substantially inhibited by TGF-ß [65]. TGF-ß has pleiotropic effects on T cell populations and plays an essential role in the maintenance of immune tolerance [65].

Another recent study revealed that in primary breast cancer, tumor cells that resist T cell attack become quiescent. Such quiescent cancer cells (QCCs) were found to form clusters (niches) with reduced immune cell infiltration [66]. A transcriptomic analysis of TILs inside and ouside such QCC niches revealed hypoxia-induced programs and identified more exhausted T cells, tumor-protective fibroblasts, and dysfunctional DCs inside the clusters. Thus, QCCs constitute immunotherapy-resistant reservoirs. These orchestrate a local hypoxic immune-suppressive milieu that blocks T cell function [66].

Like estrogen receptor positive breast cancer, prostate cancer can become undetectable after curative intent radiation or surgery, only to recur years or decades later. For induction of tumor dormancy, prostate cancer cells respond to signals from their microenvironment, including TGF-ß2, BMP-7, GAS6, and Wnt-5a. These signals result in other signals and transcription factors including SOX2 and NANOG, which likely affect the epi-genome through histone modification [67].

That signals from the microenvironment can be of relevance for the immunobiology of cancer metastasis and can lead to shifts in tumor cell phenotypes has been hypothesized as early as 1980 [68]. At that time the main hypothesis discussed in the USA was that metastasis is a result of selection of tumor cell variants pre-existing in primary tumors [69].

Dormant tumor cells appear to have similarities with cancer stem cells [70]. Genetic makeup in tumor dormancy may be pivotal but cellular context must be paramout [71].

### 3.12. TGF-ß and EMT

Cancer cells can adapt to thrive under low oxygen conditions. Studies have associated hypoxia with angiogenesis, metastasis, and chemoresistance [64]. TGF-ß is an extensive inducer of epithelial-to-mesenchymal-cell-transition (EMT) [64]. One example is retinopathy [72].

The main pathways involved in hypoxia-dependent EMT are TGF-ß, PI3K/Akt, Wnt, and Jagged/Notch. Responsible for transducing TGF-ß signals are SMAD proteins. The SMAD complex binds specific DNA regions along with transcription factors (e.g., SNAIL and ZEB) in order to modulate EMT-related gene expression [64].

### 3.13. Therapy Resistance and Cancer Hallmarks

Therapy resistance is our last topic of the TME. In 2000, Hanahan and Weinberg compiled eight key concepts with regard to cancer into hallmarks [73]: Sustained proliferative signaling, resisting cell death, deregulating cellular energetics, activating invasion and metastasis, enabling replicative immortality, inducing angiogenesis, avoiding immune destruction and evading growth suppressors. Recently, new hallmarks have been added: (i) dedifferentiation and transdifferentiation, (ii) epigenetic dysregulation, (iii) altered microbiome, (iv) altered neuronal signalling [74]. The new dimensions of cancer have been summarized by Hanahan [75].

Table 2 lists the topics discussed in chapter 3.

## 4. Counteracting Immunosuppression via Oncolytic Newcastle Disease Virus

The many features of Table 2 and the many examples of tumor-TME interactions presented demonstrate the complexity of this phenomenon. Fortunately, basic and translational research have provided many strategies on how to counteract the immunosuppressive TME. We will provide an overview focussing on the various cells of the TME and on oncolytic NDV.

### 4.1. Counteracting CAFs

It has been demonstrated that the cellular cross-talk between CAFs and cancer cells promotes OV activity [76]. TGF-ß, produced by tumor cells, reprogrammed CAFs, reduced their level of antiviral transcripts and rendered them sensitive to OV infection. In turn, CAFs produced high levels of fibroblast growth factor 2 (FGF2), initiating a signaling cascade in cancer cells that reduced retinoic acid-inducible gene I (RIG-I) expression and impeded the ability of malignant cells to detect and respond to OV infection. Furthermore, in xenografts derived from pancreatic cancer patients, the expression of FGF2 correlated with the susceptibility of the cancer cells to OV infection. An OV engineered to express FGF2 showed improved therapeutic efficacy in tumor-bearing mice compared to the non-engineered parental virus [76].

That TGF-ß produced by tumor cells can reprogram CAFs and render them sensitive to OV infection could mean enhanced replication of oncolytic NDV in the TME upon intra-tumoral application and increase of its therapeutic efficacy. In support of this conclusion, expression of RIG-I and other type I IFN responsive genes (IRF3, IFN-ß, IRF7) was reported to determine resistance or susceptibility of cells to infection by NDV [77]. Low expression of RIG-I was associated with increased susceptibility to infection [77]. Direct evidence does not yet exist, however, that NDV treatment can revert CAF-induced immunosuppression.

### 4.2. Turning Cold into Hot Tumors

#### 4.2.1. ICI Treatment

One approach to change the TME is immune checkpoint inhibition (ICI) which affects T cells. ICI can be considered at present as the most successful cancer immunotherapy in solid malignancies. In a significant proportion of treated cancer patients it appears that such treatment can turn “cold” and therapy-resistant tumors into “hot” T-cell inflamed tumors. However, even in ICI responsive tumors like malignant melanoma, a high percentage of patients remains unresponsive.

In some successfully treated patients (complete remission of tumor lesions) early tumor regression was followed by a phase where residual tumors remained dormant. The cytotoxic mechanisms of the regression phase included apoptosis, necrosis, necroptosis and immune cell-mediated cell death. To explain the dormant state, a recent review proposes immune (cytokine)-mediated induction of senescence in cancers as one important mechanism. The immune system`s ability to establish defensive walls around tumors isolate tumor cells and keeps them in a non-proliferating state [67].

#### 4.2.2. OV Treatment

OV cancer immunotherapy and the transformation of “cold” into “hot” tumors has been discussed and associated with four different types of activating the immune system: (i) Release of danger signals and DC maturation, (ii) T-cell priming and trafficking, (iii) antibody-dependent cellular cytotoxicity (ADCC) and phagocytosis and iv) T-cell/NK-cell mediated tumor cell killing [78]. Oncolytic NDV appears to change cold into hot tumors via mechanisms (i) and (iv).

**Figure 1:**
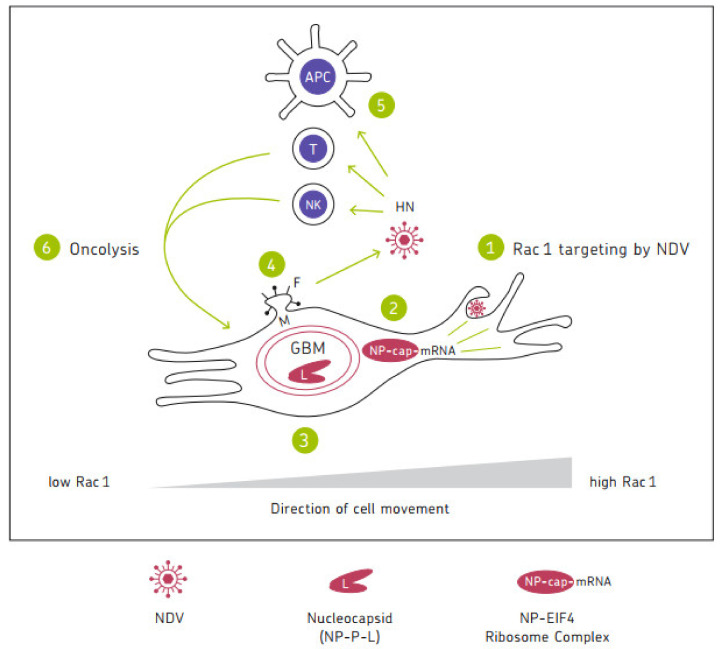
Schematic illustration of mechanisms of anti-tumor activity of NDV, exemplified with a migratory and invasive glioblastoma (GBM) cell. 1. Macropinocytosis/endocytosis of NDV targeting Rac1, which plays a cardinal role in oncogenic alterations and in development of drug resistance. The direction of cell movement is accompanied by an increase in Rac1 expression from the trailing edge (left) to the leading edge at the lamellipodia (right). 2. Targeting the cap-dependent translational machinery, viral mRNA to protein translation in the cytosol and later in the double-membraned autophagosome. This is achieved through the MNK1/2-eIF4E axis. 3. Tumor-selective virus replication in autophagosomes. The clam shaped symbol stands for the helical nucleocapsid structure composed of L, P, and NP proteins. 4. Virus progeny encapsulation, budding, and virus release mediated via M, HN, and F. 5. Antiviral response of healthy normal cells, including immune cells (APC; T, NK) initiated through recognition of HN by NKp46 and other PAMPs by TLRs and RLRs leading to IFN-I secretion and to DC1 and Th1 polarized adaptive immunity responses. 6. NDV-induced tumor cell death responses (oncolysis). These involve extrinsic (immune-mediated) and intrinsic cell death signaling pathways. ICD-derived components feed into APCs which present TAAs to T cells. Several rounds of such cycles (1–6) drive oncolytic effects and lead to immunological memory and systemic antitumor immunity. Figure taken with permission from [93].

Tumor cell infection by oncolytic NDV leads to induction of immunogenic cell death (ICD) with expression of pathogen-associated molecular patterns (PAMPs) (HN, ppp-RNA Leader, and dsRNA) and release of damage-associated molecular patterns (DAMPs) (ecto-CRT, HSP, HMGB1, and ATP) [79]. A schematic illustration of mechanisms of anti-tumor activity of NDV is shown in Figure 1 below. Further molecular details have been illustrated [80].

Pro-immunotherapeutic properties of OVs include immune activation at the tumor site and the possible effects of transgene expression [81]. Virus-based immuno-oncology models will help to further differentiate between cold vs. hot tumors. Recent reviews focus on OVs combined with bi- and tri-specific antibodies as next generation cancer immunotherapy [82,83]. Also, OVs have been developed as nanomedicines against an immunosuppressive TME [84].

Future developments of OV therapy, including genetic modification and combination therapy have been discussed [85].

#### 4.2.3. OV Treatment Combined with ICI and Role of DCs

A phase Ib clinical trial tested the impact of intratumoral OV therapy with talimogene laherparepvec (TVEC) on CTL infiltration and therapeutic efficacy by treatment of n = 21 patients with advanced melanoma with anti-PD-1 (pembrolizumab) antibody [86]. OV treatment promoted intratumoral T cell infiltration and improved the anti-PD-1 immunotherapy. The therapy was generally well tolerated and the objective response rate was 62% [86].

Intra-tumoral application of oncolytic NDV to murine melanomas had abscopal effects at sites of secondary tumors and made these susceptible to ICI therapy [87]. In the context of oncolytic NDV therapy innate immune sensing of viral RNA via RIG-I and activation of innate immune cells may enhance DC accumulation in tumors and make them more susceptible to ICI. A recent study reported that retinoic acid (the vitamin A derivative tretinoin) induces an IFN-I driven inflammatory TME, sensitizing it to ICI [88].

Apart from T cells, DCs are also important in the TME. A recent paper demonstrated that expansion and activation of CD103+ DC progenitors at the tumor site can enhance tumor responses to therapeutic PD-L1 and BRAF inhibition [89]. Adoptive T cell immunotherapy studies of human tumors in NOD/SCID mice had demonstrated that co-transfer of TAA-laden DCs supports T cell effector functions and maintenance within the treated tumor tissue [24].

### 4.3. Downregulation of MDSCs

NDV can downregulate immunosuppression in the TME. One example is the effect of antitumor vaccination by NDV pHN plasmid DNA vaccination [90]. Such vaccination at the site of the mouse ear pinna induced high levels of systemic IFN-I and reduced tumor growth in a prophylactic model of subcutaneously implanted mammary carcinoma. Analysis of the TME revealed a significant increase in NK cells and decrease in MDSCs [90].

### 4.4. Macrophage Activation and Polarization

NDV can also activate macrophages. It induces synthesis of nitric oxide (NO) and causes activation of NFkB in murine macrophages. These were part of an activation process that included stimulation of adenosine deaminase and inhibition of 5′-nucleotidase [91]. Further studies revealed that NDV-stimulated human monocytes to kill various human tumor cell lines and that the tumoricidal activity is mediated by TRAIL [92]. Soluble TRAIL-R2-Fc but not soluble CD95-Fc or TNF-R2-Fc showed a specific blocking effect. TRAIL induction on human monocytes by NDV was independent from viral replication and functioned also with UV-inactivated NDV [92]. These results suggest that oncolytic NDV in the TME can counteract M2 TAM mediated immunosuppression (see Table 2).

### 4.5. DC Activation and Polarization

Oncolytic NDV was reported to activate in immune cells a number of innate immunity sensing receptors: Protein kinase dsRNA activated (PKR), RIG-I, Toll-like receptor (TLR) and IFN-I α receptor (IFNAR) [79,93]. The effects on DCs and Th cells were priming towards DC1 and Th1 responses and thereby counteracting Th2 and Th17 polarization. In human DCs, NDV infection was demonstrated by a systems biology analysis, to upregulate within 18 h 779 genes by a choreographed cascade of transcription factors [94].

### 4.6. T Cell Activation and Polarization

T cells require for activation (i) signals via the antigen-specific T cell receptor complex TCR-CD3 as well as (ii) co-stimulatory signals via other receptors such as CD28. Infection of human melanoma cells by NDV was reported to provide co-stimulatory activity towards autologous melanoma-specific CD4+ T helper cell TILs. The co-stimulatory signals were independent of CD80/CD86 signaling [95].

The earliest report of T cell activation by NDV is from 1993. A greater than sixfold increase in peptide-specific CTL responses was observed. The findings suggested that NDV or viral HN expressed on APCs or tumor cells can exert a T-cell co-stimulatory function [96].

An experimental study, 10 years later, revealed that modification of tumor cells by a low dose of NDV could potentiate tumor-specific CD8+ CTL activity via induction of IFN-I [97]. The generation of CTL activity in vitro could be blocked specifically by antisera to IFN-I. Similar effects were observed in vivo, suggesting that IFN-I is essential for the generation of CTL activity in general [97]. IFN-I was reported to provide a third signal to CD8+ T cells to stimulate clonal expansion and differentiation [98].

Naïve T cells (Tn) require two homeostatic signals for long-term survival: TCR-pMHC contact and IL-7 stimulation. Recently, it was reported that microbial exposure has an impact on Tn homeostasis. The conversion and expansion of long-lived Ly6C+ CD8+ Tn cells depended on IFN-I, which upregulates MHC class I and enhances tonic TCR signalling in differentiating Tn cells. Moreover, for these cells, IFN-I mediated signals optimized their homing to secondary sites, extended their lifespan and enhanced their effector differentiation [99].

Upregulation of PD-L1 by various oncogenic mutations such as EGFR, BRAF, and activation of PI3K and JAK-STAT3 in tumor cells are critical pathways modulating tumor immune responses in the TME. STAT3 was recently demonstrated to contribute to oncolytic NDV-induced immunogenic cell death (ICD) in glioma, lung cancer and melanoma [100].

Whether strong T cell co-stimulation can cause re-activation of unreactive, possibly anergized MTCs from late-stage cancer patients is unknown. To investigate this, a bispecific anti-CD28 fusion protein (bsHN-CD28) was produced which can easily attach to the autologous NDV-modified tumor cell vaccine ATV-NDV. 14 colorectal carcinoma (CRC) patients with unresectable late-stage disease were treated by vaccination with the vaccine ATV-NDV to which increasing amounts of bsHN-CD28 had been attached. While no severe adverse events were recorded, all patients showed an immunological response of tumor-reactive MTCs, at least once during the course of five vaccinations. A dose-response relationship of the response with the amount of co-stimulatory protein was seen. A partial response of metastases was documented in four patients. The study suggests that the three-component vaccine is safe and can re-activate possibly anergized T cells from a chronic disease like advanced-stage cancer [101].

### 4.7. NK Cell Activation

Upon infection by oncolytic NDV, human carcinoma and melanoma cells showed enhanced expression of ligands for the NK cell cytotoxicity receptors NKp44 and NKp46 [102]. The HN protein of NDV served as ligand. NKp44- and NKp46-CD3zeta lacZ-inducible reporter cells were activated by NDV-infected tumor cells. NDV-infected tumor cells stimulated NK cells to produce increased amounts of the effector lymphokines IFN-γ and TNF-α. NK cell lysis of NDV-infected tumor cells was eliminated by the treatment of target cells with the neuraminidase inhibitor Neu5Ac2en. These results suggested that direct activation of NK cells contributes to the antitumor effects of NDV [102].

Further studies revealed that HN, upon interaction with NKp46 receptor, upregulates expression of the tumor necrosis factor-related apoptosis inducing ligand (TRAIL)-death receptor (TRAIL-R) in murine NK cells through activation of spleen tyrosine kinase (Syk) and nuclear factor kappa B (NFkB) [103]. Exposure of NK and T cells to NDV resulted in enhanced tumoricidal activity that was mediated by upregulated TRAIL via an IFN-γ dependent pathway [103].

### 4.8. Targeting Rac1 and Spread of NDV in Tumors

#### 4.8.1. Targeting Rac1

Oncolytic NDV exerts anti-neoplastic as well as immune stimulatory properties. Molecular mechanisms of these dual properties have recently been elucidated [93]. One important aspect of the viruses anti-neoplastic activity is the targeting of migratory cancer cells via the Rho GTPase Rac1 [104]. Figure 1 shows a schematic illustration of a migratory and invasive glioblastoma cell. The direction of cell movement is accompanied by an increase in expression of Rac1 at the leading edge of the lamellipodia. NDV targets Rac1 at the lamellipodia via macropinocytosis/endocytosis. Following cell entry, NDV targets the cap-dependent translational machinery through the MNK1/2-eIF4E axis [93]. Tumor-selective virus replication then occurs in autophagosomes [56]. F and HN proteins play important roles in virus release and immune cell activation. Oncolysis is exerted by ICD [93].

#### 4.8.2. Spread of Oncolytic NDV in Tumors

Virus spread in tumors starts with virus progeny encapsulation, membrane budding and virus release mediated via M, HN and F [93]. The release of progeny virions from the surface of infected cells is facilitated by neuraminidase activity located at the sialidase ß-propeller domain of HN [92]. Another important virus protein for spread in tumors is the fusion protein F. Mutations at the cleavage site of the F precursor protein F_0_ facilitate multicyclic virus replication. The formation of syncytia by fusogenic NDV strains leads to virus spread to neighbouring cells and reduces exposure to virus neutralizing antibodies [93]. NDV particles produced in autophagosomes can possibly be exported from the cell via a process designated as secretory autophagy [105].

NDV infection of stem-cell enriched spheroids of lung cancer inhibited their 3D growth potential in vitro. The infection resulted in the degradation of LC3 and P62, two hallmarks of autophagy maturation. Apparently, NDV promoted autophagy flux in these spheroids as confirmed by transmission electron microscopy [93].

NDV taken up in Rab5a positive endosomes or macropinosomes can be recycled for export via exosomes [106]. This would increase viral spread in tumor tissue. Rab5a was found to be associated with genes involved in exosome secretion [107]. NDV-related exosomes containing NP proteins and microRNAs were reported to exhibit replication-promoting and IFN-ß-suppressing abilities [108,109].

### 4.9. Suppression of the Metabolic Barrier

The TME also represents a metabolic barrier (Table 2). Cancer cells increase glycolysis rates to generate ATP as the primary energy source for cell growth and proliferation. A recent study now revealed that infection of human breast cancer cells with oncolytic NDV suppresses the glycolysis pathway. The NDV exposed cancer cells in contrast to normal embryonic REF cells showed decreased hexokinase activity, decreased pyruvate and ATP concentrations and decreased acidity. NDV infection induced cell death (oncolysis) in the cancer cells but not in the normal control cells [110].

Blockade of the immunosuppressive oncometabolic circuity has been reported by application of the oncolytic adenovirus Delta-24-RGDOX in combination with IDO inhibitors [111]. Another recent study reports that localized delivery of PD-1 inhibitors by engineered oncolytic herpes simplex virus (YST-OVH) activates collaborative intratumoral response to control tumor and synergizes with CTLA-4 or TIM-3 blockade [112].

### 4.10. Breaking Cancer Therapy Resistance and Immune Resistance

#### 4.10.1. Breaking Resistance to Chemo- or Radiotherapy

Oncolytic NDV is a cancer-therapeutic biological agent that, in contrast to chemotherapy and radiotherapy, does not require cells to be in a proliferating state. The RNA virus replicates in the cytoplasm of cells and is independent of DNA replication. NDV can replicate in X-irradiated cells (e.g., ATV-NDV vaccine) because X-irradiation damages DNA but not RNA. As a consequence, oncolytic NDV can potentially replicate and destroy tumor cells in a resting state such as tumor stem cells or cells from tumor dormancy that may not be affected by chemo- or radiotherapy. This suggests that oncolytic virus therapy with NDV may well complement conventional cancer therapies [113].

#### 4.10.2. Breaking Resistance to Hypoxia, Apoptosis, TRAIL, Drugs and Small Molecule Inhibitors (SMIs)

The center of a growing tumor is often hypoxic. A hypoxic TME induces the transcription factor HIF which influences gene expression and contributes to tumors radio-, and chemo-resistance. It was reported that oncolytic NDV can break resistance to hypoxia [114].

Oncolytic NDV can also break resistance to apoptosis and TRAIL. An increased oncolytic activity was reported utilizing a human non-small-cell lung cancer cell line overexpressing the anti-apoptotic protein Bcl-xL. The enhanced oncolytic activity was secondary to enhanced viral replication and syncytium formation [115]. A similar result was observed with apoptosis-resistant primary melanoma cells that overexpressed the inhibitor of apoptosis protein Livin [116]. Caspases could cleave Livin to create a truncated protein with proapoptotic activity [116]. TRAIL-resistant hepatocellular carcinoma-derived cell lines were found to be more susceptible to NDV-mediated oncolysis than TRAIL-sensitive cells. IFN-stimulated gene-12a over-expression or silencing enhanced or reduced the cells TRAIL sensitivities [117].

Recent treatment strategies incorporating ICIs and anti-angiogenic agents have brought many changes and advances in clinical cancer treatment [118]. However, challenges still exist with regard to immune suppressive tumors, which are characterized by lack of T cell infiltration and treatment resistance. Crosstalk between angiogenesis and immune regulation in the TME has been recently reviewed [118].

Rac1 signaling has been identified as a major mediator of drug-resistance mechanisms [119]. Examples are v-raf murine sarcoma viral oncogene homolog B (BRAF) protein inhibitors in melanoma. Their effectivity is restricted by resistance mechanisms. Under NDV mediated hyper-activation of Rac1, Rac1-GTP activates Pak1, leading to the downstream activation of mitogen-activated protein kinase (MEK) and to bypassing BRAF inhibition [119].

#### 4.10.3. Breaking of T Cell Tolerance to TAA-Expressing Tumor Cells

NDV infection of human melanoma cells could break tolerance of a melanoma-specific CD4+ T helper cell line [96]. Potentially anergized TAA-specific T cells from late-stage CRC patients could become re-activated by strong T-cell costimulation involving NDV and anti-CD28 mediated signals [101].

In tumor immunology, the concept of augmentation of costimulatory signals in T cells is complementary to that of inhibition of negative signals delivered via coinhibitory checkpoint receptors.

#### 4.10.4. Breaking Resistance to Oncolysis, to ICI and to Anti-Viral Immunity

Intra-tumoral application of NDV to murine melanoma [86] and to oncolysis-resistant bladder cancer [120] had abscopal effects at sites of secondary tumors and made these susceptible to ICI therapy. Oncolysis-independent immune stimulatory effects of NDV are based on increased adhesiveness mediated by HN [92].

Anti-viral immunity is considered as a major hurdle for effective therapeutic avtivity of OVs. With regard to NDV, it was reported from an animal model that pre-existing anti-viral immunity potentiated rather than inhibited its immunotherapeutic efficacy [121].

Chapter 4.10. has summarized evidence that oncolytic NDV has the potential to break therapy resistance and immune resistance. It is the first OV for which such potential has been reported. This explains why the authors focus on NDV.

### 4.11. Recruitment of Cancer-Reactive MTCs

Spontaneous anti-tumor T cell responses in the BM against blood-borne antigens, including TAAs have been described as well as the presence of cancer-reactive MTCs in the BM of cancer patients (Table 1). The recruitment of such cells from the BM to the site of a primary tumor or a metastatic site by active-specific vaccination can be expected to exert a tumor-protective effect.

One of many clinical studies supporting this concept was performed in colorectal carcinoma (CRC) patients. CRC is one of the leading causes of cancer-related deaths worlwide. Surgery remains the primary curative treatment but nearly 50% of patients relapse as consequence of micrometastatic or minimal residual disease (MRD) at the time of surgery.

A prospective randomized trial investigated the efficiency of adjuvant active-specific immunization (ASI) with autologous NDV modified tumor vaccine (ATV-NDV) in stage IV CRC patients as a tertiary prevention method following resection of liver metastases [122]. 50 patients were available for analysis after a long follow-up period of about ten years. While rectal carcinoma patients apparently did not profit from this type of immunotherapy, a subgroup analysis revealed a significant advantage for vaccinated colon cancer patients with respect to overall survival (OS) and metastases-free survival [122]. In the control arm, 78.6% had died, in the vaccinated arm only 30.8%. The trial provides clinical evidence for the value and potential of the cancer vaccine ATV-NDV.

The observed improvement of long-term survival was explained by activation and mobilization of a pre-existing repertoire of cancer-reactive MTCs [123] which reside in distinct niches of patient`s bone marrow [14]. This is possible by the use of an autologous cancer vaccine. MTC niches are in neighbourhood with hematopoietic (HSC) and mesenchymal (MSC) stem cells. BM MTCs also contain a subset of stem memory T cells (SMTs) in addition to effector (EMTs) and central memory T cells (CMTs). The recruitment of cancer-reactive BM MTCs to the site of vaccination with ATV-NDV occurs through recognition of TAAs in association with NDV-induced pro-inflammatory cytokines and chemokines [123].

### 4.12. In Situ Vaccination

In situ vaccination against cancer is a concept that is being developed based on mechanisms of immunogenic cell death, ICD, and on DC vaccination. Knowledge about DC vaccine`s therapeutic value has been increased in the past decade. Improvements of this “advanced therapy medicinal product (ATMP)” have been achieved. For instance, a superior monocyte-derived DC preparation has been reported. It includes short-term culture with IL-15, pro-inflammatory cytokines and immunological danger signals. In situ silencing of programmed-death ligands potentiated anti-tumor potency of such a DC vaccine [124].

#### 4.12.1. Photothermal Treatment Inducing ICD

Recent studies have discovered that certain immunotherapeutic photosensitizers, such as Rose Bengal (RB) can improve antitumor immune responses by triggering ICD. “Eat me” and “danger” signals such as calreticulin (CRT) become expressed on the surface of tumor cells undergoing ICD. They can enable immature DCs (iDCs) to phagocytose tumor cells and present TAA epitopes to T cells through MHC-I or -II molecules. A novel in situ DC vaccine triggered by RB was reported to enhance adaptive antitumor immunity [125].

Local tumor photothermal treatment with near-infrared light (NIR-II) is a promising strategy in triggering in situ tumor vaccination for cancer therapy, in particular of skin pre-cancerous lesions. However, limited penetration of photothermal agents within tumors seriously limits their spatial effects. In a recent study, a deep tumor penetrating gold nano-adjuvant is described which significantly inhibits tumor growth, induces a cascade of immune responses, generates adaptive immunity against the re-challenged cancers and boosts an abscopal effect which completely inhibits pulmonary metastases [126].

#### 4.12.2. IMI Strategy

A new individual multimodal cancer immunotherapy (IMI) strategy has been developped at IOZK (Cologne, Germany) [127]. It combines (i) local moderate electrohyperthermia (mEHT)/ oncolytic NDV pre-treatment for in situ vaccination with (ii) specific autologous anti-tumor vaccination employing the ex vivo generated DC vaccine IO-VAC^R^ (formerly termed VOL-DC). IO-VAC^R^ is a ATMP product consisting of a modern DC vaccine pulsed with patient-derived NDV oncolysate. In the first step, the patient’s immune system is conditioned by NDV towards a Th1 polarized immune response based on in situ induction of ICD combined with locally applied mEHT. Some results from this IMI therapy will be presented and discussed under 5.1.

#### 4.12.3. Use of GM-CSF Modified NDV

The murine gene encoding GM-CSF was inserted in 2007 as an additional transcription unit at two different positions into the NDV genome. The recombinant virus rNDV-muGM-CSF with the strongest production of the transgene product was selected for further studies. Tumor vaccine cells infected with rNDV-GM-CSF stimulated human PBMCs to exert antitumor bystander effects in vitro in a tumor neutralization assay (TNA). These effects were significantly increased compared to rNDV without the transgene. In addition, rNDV-GM-CSF led to a much higher IFN-I production in PBMC than rNDV when added as virus or as virus-modified vaccine. Monocytes and plasmacytoid DCs were demonstrated to contribute to the augmented IFN-α response. Thus, the already inherent anti-neoplastic and immunostimulatory properties of NDV could be further augmented. The transgene product initiated the recruitment of DCs and a broad cascade of immunological effects [128].

A recent manuscript reports that a similar recombinant NDV (rNDVhuGM-CSF (MEDI5395 from Astra Zeneca)) combined broad oncolytic activity with the ability to modulate genes related to immune functionality in human tumor cells [129]. Intratumoral injection conferred antitumor effects in three syngeneic models in vivo. The efficacy could be further augmented by concomitant treatment with anti-PD-1/PD-L1. Ex vivo immune profiling, including TCR sequencing, revealed profound changes consistent with priming and potentiation of adaptive immunity and TME reprogramming toward an immune permissive state [129]. A clinical study (NCT03889275) is in progress.

### 4.13. Use of Antibody Modified NDV

It was demonstrated that a recombinant NDV could express a full IgG antibody from two transgenes [130]. The antibody targeted an angiogenesis epitope on vascular endothelial cells of the TME. The use of antibody-modified NDV allows to combine the advantages of oncolytic RNA viruses and monoclonal antibodies in a single powerful anticancer agent. In fact, oncolytic NDV expressing a chimeric antibody against the TAA CD147 enhanced anti-tumor efficacy in orthotopic hepatoma-bearing mice [131].

### 4.14. Altering the TME by Sytemic Transfer of OV-Loaded Carrier Cells

Activated T cells can be loaded with oncolytic NDV in such a way that the virus load can be transferred to tumor target cells upon contact of the virus loaded T cells with tumor cells [132]. NDV “hitchhiking” could potentially increase NDV tumor targeting after systemic transfer of virus loaded T cells [132].

Oncolytic NDV could also be delivered to tumors by BM derived MSCs [133]. OV delivery by MSCs enhanced therapeutic effects altering the TME [133].

### 4.15. Active-Specific Immunization (ASI)

Prevention of metastatic spread was reported by post-operative active-specific immunization (ASI) with NDV modified but not with unmodified irradiated autologous tumor cell vaccine [134]. About 50% of the mice immunized with NDV-modified vaccine survived longterm while mice immunized with unmodified vaccine were dead within three weeks. Post-operative activation of tumor-specific CTL precursors (CTLPs) from mice with metastases required stimulation with the specific TAA plus additional signals. Such signals could be provided by NDV and resulted in the augmentation of CD4+ T helper and CD8+ CTLP T-T cell cooperation [135]. These findings provide a mechanistic explanation for the in vivo effect of NDV modified vaccine.

Associated studies provided early evidence for the generation of protective immune T cell-mediated memory responses to cancer [136].

Results from clinical ASI studies employing the vaccine ATV-NDV have been presented under 4.10.

ASI can be well combined with ICI therapies.

### 4.16. Adoptive Cellular Immunotherapy (ACT)

Successful examples of this approach have been presented in Chapters 2.3 to 2.5. Effective immune rejection was demonstrated in a GvL animal model of advanced leukemia (2.5.). Human acute leukemias are also responsive to allogeneic ACT. It requires clinics which are specialized to deal with problems related to graft-versus-host disease (GvHD) and host-versus-graft (HvG) reactivity.

A few selected publications from 2022 demonstrate further novel approaches of ACT: (i) adoptive immunotherapy with engineered invariant natural killer T (iNKT) cells to target cancer cells and the suppressive microenvironment [137]. Off-the-shelf third-party HSC-engineered iNKTcells were demonstrated in xenograft models to ameliorate GvHD while preserving a GvL effect in the treatment of blood cancers [138]. (ii) use of IL15 in cell-based cancer immunotherapy [139], (iii) immunotherapy of TAA positive common solid cancers with natural high-avidity TCR-engineered T cells [140], (iv) improvement of immunotherapeutic efficacy against solid tumors in mice with dual-specific chimeric antigen receptor (CAR) T cells expanded in vitro with TCR reactivity against OV encoded antigens [141]. Illustrations of the fight of T cells against tumor cells can be seen from several references of this publication [2,11,13,23,28,56,113,137].

In GBM patients, T cells can be activated in situ by vaccination. Another procedure is adoptive T-cell immunotherapy (ACT). One study reported cross-talk between T cells and bone marrow hematopoietic stem and progenitor cells (HSPCs) during ACT in vivo in GBM hosts [142]. Transfer of HSPCs with concomitant ACT led to the production of activated CD86+CD11c+MHC-II+ cells consistent with a DC phenotype and function within the brain TME. These cells relied on T-cell-released IFN-γ to differentiate into DCs, activate T cells, and reject intracranial tumors [142].

### 4.17. Tumor-Suppressing Functions of Immune Cells within the TME

Tumor-associated immune cells within the TME with tumor-suppressing function include NK cells, DCs, M1 TAMs and effector T and B cells. The latter kill cancer cells by granule exocytosis and FasL-mediated apoptosis induction, polarizing M2-TAMs to M1 TAMs and inducing DC maturation. Effector B cells produce Th1 cytokines, enhance CTL activity and NK cell-mediated tumor cell killing [143]. While tumors can polarize DCs, macrophages and T cells from the TME towards immunosuppression, immunotherapeutic strategies can change the polarization of these cells towards a tumor-suppressing effect.

Table 3 lists the various aspects of counteracting immunosuppression in the TME via oncolytic NDV.

## 5. Post-Operative Vaccination with or without NDV of Glioblastoma Patients

Having presented evidence for spontaneous anti-tumor T cell responses in animal models and cancer patients, of immunosuppression by the TME and of the counteracting potential of oncolytic NDV, this Chapter considers one fatal cancer disease of the brain, GBM, to explore by some examples where we stand at present with regard to immunotherapeutic strategies.

The brain TME contains numerous distinct types of non-neoplastic cells which serve a diverse set of roles relevant to the formation, maintenance, and progression of central nervous system cancers [144,145]. T cells in brain TME are low in numbers and characterized by tolerance, ignorance, anergy, and exhaustion. Distinct exhaustion profiles have been reported for GBM TILs in comparison to peripheral blood T cells [146].

Despite apparent challenges, such as the blood-brain barrier (BBB) composed of tight-junction endothelial cells and astrocyte endfeet, some endogenous T cells are nevertheless capable of infiltrating GBM. A recent study identified T cell subsets expressing the chemokine receptors CCR2, CCR5, CXCR3, CXCR4, CXCR6 and the integrin-adhesion molecules CD49a and CD49d as being enriched in GBM tumors compared to matched peripheral blood T cells [146].

### 5.1. Vaccination Studies with NDV

Reasons for selection of GBM are as follows: (i) sytemic application of NDV to GBM patients has resulted, in single-case studies, to impressive results [147,148], (ii) apparently, NDV could pass the blood-brain barrier after systemic application [148], (iii) Rac1 targeting by NDV at the invasion front of migratory GBM cells [93,104].

The studies selected in Table 4 are based on T-cell based immunotherapeutic principles, including DCs as APCs and pMHC complexes as TAAs. They are post-operative vaccination studies without or with oncolytic NDV support.

The earliest type of such a study is from 2004 [149]. Operated adults with primary GBM were treated post-operatively for ASI with the already mentioned autologous tumor-cell vaccine ATV-NDV, prepared from patient-derived primary tumor cell cultures. 23 such patients were compared in this non-randomized study to 87 similar non-vaccinated patients treated at the same institution and during the same time period by standard therapy. 91% of vaccinated patients survived 1 year, 39% survived 2 years (compared to 11% in the control group), and 4% were long-term survivors. In the vaccinated group, immune monitoring revealed significant increases (i) of delayed-type hypersensitivity (DTH) reactivity of the skin towards autologous tumor cells, (ii) of numbers of tumor-reactive MTCs in peripheral blood and (iii) of numbers of CD8+ TILs in secondary tumors. The conclusion was that post-operative vaccination with ATV-NDV was feasible and safe and appeared to improve the prognosis of patients with GBM [149].

The second study describes a new strategy of cancer immunotherapy combining hyperthermia/oncolytic NDV pre-treatment with specific autologous anti-tumor vaccination employing the DC vaccine IO-VAC^R^ [127]. The first Kaplan-Meier analysis of 10 treated GBM patients revealed a median OS of 30 months. This can be compared to a median OS of 14.6 months with standard radio/chemotherapy according to the Stupp protocol [127].

The third study describes the induction of ICD during maintenance chemotherapy and subsequent IMI for GBM [150]. This retrospective analysis of 60 adults with primary GBM treated at IOZK suggested that the additional induction of ICD via NDV/mEHT during temozolamide maintenance (TMZm) cycles is beneficial in improving OS [150].

The prognosis of GBM patients with isocitrate dehydrogenase 1 (IDH1) wild-type MGMT promoter-unmethylated remains poor. All adults meeting these criteria and treated from 06/2015 to 06/2021 at IOZK were selected for a retrospective analysis [151]. Goup 1 patients (n = 9) were treated with surgery/radio(chemo)therapy and susequently with IMI. Group 2 patients (n = 25) were treated with radiochemotherapy followed by TMZm plus IMI during and after TMZ. The mean OS of group 1 patients was 11 months while that of group 2 patients was 22 months with a two-year OS of 36%. The difference was significant with a Log-rank p of 0.0001. The conclusion was that a synergy between TMZ and IMI had improved OS in group 2 patients [151].

In another retrospective analysis from IOZK involving n = 70 GBM patients, the 2-year OS was 38.8 %. When stratified for MGMT promoter methylation status, there was a highly significant difference [152].

One particular innovative finding in study [152] was a case of complete remission and specific T cell response. This case is presented with some detail to demonstrate the individuality and multimodality of the treatment process. An 18-year-old patient had an incomplete resection of a left frontal lobe IDH1 wild-type and MGMT unmethylated GBM. The tumor mutational burden was low (0.5 variants/megabase), and there was no evidence for microsatellite instability or germline variants. At presentation her Karnofsky performance index was 90. She was lymphopenic with low NK cell functioning and she had Th2/Th17 skewing. The treatment consisted of radiotherapy and, subsequently, five cycles of TMZm chemotherapy. She continued treatment for another seven TMZm chemotherapy cycles combined with 5-day ICD treatments, which were given during each TMZ cycle at days 8 to 12. Afterwards, she received two IO-VAC^R^ DC vaccinations. The DCs were loaded with ICD-treatment induced, serum-derived, antigenic extracellular microvessels, and apoptotic bodies. Later on, she received two IO-VAC^R^ vaccines loaded with tumor-specific peptides based on the individualized tumor-specific neo-antigen detection test performed at CeGaT (www.CeGat.de). She underwent complete remission. From the T cell response analysis it was clear that surgery, radiochemotherapy, and five cycles of TMZm did not induce a tumor-specific T-cell response. However, the addition of seven ICD treatments to the last seven TMZm treatments and the first IO-VAC^R^ vaccine generated a clear tumor-antigen-specific CD4+ and CD8+ T-cell response. The response was further boosted by the DC vaccines loaded with tumor-specific peptides. This observation has important impacts: (i) it is not mandatory any more to have fresh frozen tumor material to prepare a tumor lysate as a TAA source, (ii) ICD treatments allow to yield TAAs that are expressed within the body (e.g., serum) at the time of treatment [152].

Brain tumors, unfortunately, can also affect children. The prognosis of children with diffuse intrinsic pontine glioma (DIPG) remains very poor despite radio- and chemotherapy or SMIs. Surgery is no option. IOZK reported recently about a single institution experience with IMI involving n = 41 children with DIPG [153]. When IMI was part of primary treatment, median PFS and OS were 8.4 m and 14.4 m from the time of diagnosis, respectively, with a 2-year OS of 10.7%. It was concluded that multimodal immunotherapy for these children is feasible without major toxicity [153].

### 5.2. Vaccination Studies without NDV

High-grade gliomas (HGG) have an incidence currently estimated at 14,000 new diagnoses per year, according to the 2007 WHO classification, which includes patients with anaplastic astrocytomas (WHO grade III) and with GBM (WHO grade IV). To evaluate the therapeutic efficacy of TAA-pulsed DC treatment, a systematic analysis in terms of patient survival of relevant published clinical studies was performed [154].

An electronic search yielded 189 references. From these, 9 articles were selected for reasons presented. A total of 409 patients, including historical cohorts, nonrandomized and randomized controls with HGG, were the basis of the meta-analysis. DCs were matured using cocktails containing GM-CSF, IL-4, TNF-α, IL-1ß, or PGE2. The sources of TAA were different. Most were derived from tumor cells: autologous irradiated tumor cells, autologous tumor lysate, HLA-I-eluted peptides, autologous acid-eluted tumor peptides and autologous heat-shock tumor cells. The routes of DC injection were mainly intradermal, intratumoral or subcutaneous. The numbers of TAA-pulsed DCs injected ranged from 1 × 10^6^ to 5 × 10^8^.

Here we present 2-year OS data from seven of those trials (with n = 354 patients). The OS rates were 34% for HGG patients receiving DC treatment and 14% for the controls. The difference was highly significant (*p* < 0.00001) [154].

Comparisons were also made for 3-, 4-, and 5-year OS between the non-DC and DC groups in HGG patients using Forest plot analysis. The odds ratios (OR) all favoured the DC vaccination group [154].

A recent Nature article reports about a T helper type peptide vaccine targeting mutant IDH1 in newly diagnosed glioma [155]. Mutated IDH1 defines a molecularly distinct subtype of diffuse glioma. Pre-clinical studies had demonstrated that a specific peptide vaccine (IDH1-vac) induces specific therapeutic Th responses that are effective against IDH1 (R132H) mutant tumors in syngeneic MHC-humanized mice. A multicentre, single-arm, open-label, first-in-humans phase I trial was carried out in 33 patients with newly diagnosed WHO grade 3 and 4 IDH1 (R132H)+ astrocytomas (NCT02454634). Vaccine-induced immune responses were observed in 93.3% of patients across multiple MHC alleles. Three-year progression-free and death-free rates were 63 and 84% respectively. Pseudogrogression was observed at high frequency and associated with increased vaccine-induced T cell responses. The three-year survival rate of 84% is impressive but cannot be compared with the previous GBM studies, since 2/3 of the patiens were astrocytomas grade 3 [155].

Table 4 contains a list of the mentioned GBM studies.

**Table 4:** Post-operative vaccination with or without NDV of glioblastoma patients.

Feature	Vaccine Type/Patients	Year	Comments	References
ASI	ATV-NDV Adults with primary GBM (n = 23)	2004	Two-year survival rate 39% compared to 11% in control group	[149]
IMI	IO-VAC^R^ Retrospective analysis of adults with primary GBM (n = 10)	2017	Combining NDV with DC vaccination and mEHT	[127]
ICD	IO-VAC^R^ Adults with primary GBM (n = 60)	2018 2021 2022	Combining IMI with chemotherapy, IDH1 wt (n = 25)	[150] [152] [151]
IMI ICD Peptides+	IO-VAC^R^ + peptides (n = 1)	2021	A case of CR and specific T cell response	[152]
IMI	IO-VAC^R^ Children with DIPG (n = 41)	2020	A single institution experience	[153]
Meta analysis	TAA pulsed DC vaccine without NDV	2014	Clinical efficacy	[154]
Th peptide vaccine	IDH1-vac, Adults with astrocytomas grade 3 (n = 22) and 4 (GBM) (n = 11), Targeting mutant IDH1	2021	Three-year survival rate 84%, no control group	[155]

ASI = Active-specific immunotherapy; ATV-NDV = Autologous Tumor cell Vaccine modified by infection with NDV; DC = Dendritic cell; DIPG = Diffuse intrinsic pontine glioma; GBM = Glioblastoma multiforme; ICD = Immunogenic cell death; IDH1 wt = isocitrate dehydrogenase wildtype; IDH1-vac = a peptide vaccine based on mutated IDH1; IMI = Individual Multimodal Immunotherapy; IO-VAC^R^ = DC vaccine pulsed with NDV oncolysate; mEHT = moderate electrohyperthermia.

## 6. Conclusions

Avoiding immune destruction is one of the hallmarks of cancer. To achieve this, cancer cells interact with host cells and organize an immunosuppressive tumor microenvironment. One important factor in this context is TGF-ß. It is secreted by tumor cells and regulatory T cells (Tregs), affects cancer-associated fibroblasts (CAFs), macrophages (M2-TAM) and NK cells. In addition it is involved in the formation of the pre-metastatic niche and in epithelial to mesenchymal cell transition (EMT) (Table 2). Tumor-promoting immune cells of the TME include among others M2-TAMs, Tregs and MDSCs.

This review proposes to employ the avian oncolytic virus NDV and cellular immunotherapy to counteract the immunosuppressive influence of the TME. NDV is the first OV with reported potential to break therapy resistance, drug resistance and immune resistance (see Chapter 4.10.).

Intratumoral inoculation of NDV can counteract immunosuppression by inducing a strong IFN-I response and by activating innate and adaptive immunity systems. In addition to its immune stimulatory activities, NDV exerts a variety of anti-neoplastic functions such as tumor-selective oncolysis and breaking several types of resistancies.

In contrast to the TME, the microenvironment of the bone marrow favors spontaneous anti-cancer immune responses. Tumor-induced angiogenesis connects a locally growing tumor with the blood circulatory system thereby releasing tumor cells and TAAs into the blood. There is a bi-directional connection between blood and BM. All cellular components of the blood are derived from hematopoietic stem cells (HSCs) of the BM. The BM parenchyma contains among others resident CD11c+ DCs. These capture blood-borne antigens, including TAAs, process them and present them to CD4+ and CD8+ T cells arriving from the blood through BM sinuses.

A distinct speciality of the adaptive immunity system with importance for the fight against cancer and its metastases is its memory function. Memory T cells consist of various subtypes and represent a very dynamic system of control. BM plays an important role in memory homeostasis and provides distinct niches for maintenance and long-term survival of MTCs. Powerful MTCs could be generated in mice from a naïve T cell repertoire against a surrogate TAA. Upon transfer to tumor cell challenged T cell deficient mice, these MTCs protected the mice and thereafter returned into a resting state in niches from the BM. From there, they could be re-activated via antigenic challenge and recruited into the peritoneal cavity. Transfer of these peritoneal MTCs to secondary tumor cell challenged T cell deficient hosts again protected the mice. Four such successive transfers provided evidence for longevity and functionality of TAA-reactive CD8+ MTCs.

Spontaneously induced cancer-reactive MTCs have been documented to exist in the BM of patients with different types of cancer. Their mobilization and recruitment to the site of a tumor would be another way of counteracting an immunosuppressive TME. Post-operative active-specific immunization of cancer patients with autologous tumor vaccines modified by oncolytic NDV was apparently capable to mobilize and recruit cancer-reactive MTCs from the BM and/or from other tissue sites. An example is colon cancer (stage IV), where a randomized-controlled clinical study revealed a long-term survival benefit by about 30% of thus vaccinated patients.

Cellular immunotherapies involving transfer of allogeneic MHC-matched immune T cells (GvL model) or re-activated human MTCs from the BM of cancer patients to tumor-bearing NOD/SCID mice (tumor xenotransplant model) led to infiltration of tumors by the T cells and to tumor rejection.

An individualized multimodal immunotherapy strategy and protocol has been established at IOZK, Cologne, Germany. It employs systemic oncolytic NDV in combination with local mEHT in a pretreatment phase to polarize the patient’s immune system towards DC1 and Th1 responses. The NDV induced IFN-I was recently reported to prime a subtype of DCs (ISG+DCs) to acquire and present whole pMHC complexes. Following the pretreatment phase, patients receive active-specific immunization with IO-VAC^R^, a patient-derived DC vaccine pulsed with NDV-mediated oncolysate.

One fatal cancer of the brain, GBM, has been selected in this review to demonstrate what type of result can be obtained with immunotherapeutic approaches. The TME of GBM represents a particular challenge because of strong immunosuppression. Accordant results obtained in several GBM phase I/II studies including a meta-analysis of 354 high grade glioma patients vaccinated with DC vaccine suggest that anti-tumor vaccination has the potential to prolong overall survival.

Another recent experimental study in GBM patients reported on cross-talk between adoptively transferred T cells and BM derived hematopoietic stem cells. This led to the production of activated DCs within the brain TME.

It can be concluded that immune cells within the TME with immunosuppressive functions (e.g., NK cells, DCs, TAMs, T and B cells) can be converted to immune cells with tumor-suppressing function. This can be achieved locally (e.g., by intratumoral application) or systemically. The review provides examples of both approaches including OVs and cellular immunotherapy.

The examples include important human cancer types, such as breast cancer (2.2., 4.3., 4.9.), pancreatic cancer (2.2.), renal carcinoma (3.11.), prostate carcinoma (3.11.), melanoma (4.2.3.), lung cancer (4.8.), colon carcinoma (4.11.), gastric cancer (4.17.), and glioblastoma multiforme (5.). Also bone marrow or blood derived cancers (leukemias) respond to these types of immunotherapy (2.5., 4.15.).

A few decades ago it was heavily disputed whether the immune system might have anything to do with cancer, especially with cancer in human. Meanwhile it can be concluded: the mere fact that tumors need an immunosuppressive microenvironment to grow is evidence for a role of immunosurveillance in cancer. Progress in research of molecular, cellular and tumor immunology has led to several Nobel prizes and to immunology-based therapeutics, such as antibodies (mAbs, including ICI reagents, and bs-Abs), cancer- and DC-vaccines, CAR-T cells and others. Such immunotherapeutics represent a change of paradigm in the treatment of cancer.

The results presented demonstrate the importance of innovative experimental and translational studies to improve effective therapeutic treatments of cancer patients. Examples of the review have demonstrated that the potential of the immune system to fight cancer goes far beyond what present-day cancer immunotherapy achieves. So it is worth to invest more research effort into this direction.

## Figures and Tables

**Table 1:** Spontaneous anti-tumor T cell responses.

Feature	Mol Determinant	Year	Comment	References
MHC-I TAA cross-presentation	mRNA m^6^A, YTHDF1 protein	2019	CD8+ T cell response after immunoediting in DCs	[15]
MHC-II neoantigens	hmMHC binding predictor	2019	CD4+ T helper cell responses, a key function in therapy	[17]
DC1	APC function IFN-I BM DCs	2022 2008	ISG+ CD11b+ DCs Resident, APC function	[16] [4]
BM as priming site against blood-borne TAAs	ß-galactosidase (lacZ) and ovalbumin as surrogate TAAs	2003 2003	CD8+ T cell response in BM, potential for long-lasting protective anti-tumor immunity	[4] [11]
Dormant tumor cells from BM	Maintenance of TAA-specific MTCs	2003 2005	Adoptive MTC transfer in nude mice	[5] [6]
Breast cancer	BM MTC repertoire	2006	Polyvalent and highly individual	[19]
Pancreatic cancer	Functional BM MTCs	2005	High frequencies	[21]
Solid tumor patients	WT1-specific CD8+ T cells	2010	High frequencies BM as secondary lymphoid organ	[12]
Therapy of human tumors	Re-activated BM MTCs from breast cancer, Clinical study	2001 2003 2004 2009 2013	Adoptive MTC transfer in NOD/SCID mice Expansion capacity Overall survival	[23] [22] [24] [26] [27]
Rejection of advanced metastasized cancer	Vß6 donor T cells recognizing tumor-associated vSAG7	1995	Adoptive transfer of allogeneic MHC identical immune T cells	[28]

ATV-NDV = Autologous Tumor cell Vaccine modified by infection with NDV; BM = Bone marrow; bsAb = bispecific antibody; DC = Dendritic cell; MHC = Major histocompatibility complex; MTC = Memory T cell; TAA = Tumor associated antigen; WT1 = Wilms tumor 1 antigen; vß6 T cells = T cells expressing TCR vß chain 6; vSAG7 = a viral superantigen from murine endogenous mammary tumor virus (MMTV).

**Table 2:** The tumor microenvironment.

Feature	Mol Determinant	Year	Comment	References
3.1. BM TME	Targeting bone metastases	2022	Novel approaches	[33]
3.2. CAF	ECM, cytokines, TGF-ß, PAI-1, EMT	2022	Increase in cancer cell motility, proinvasion and proangiogenic factors	[38]
3.3. TME “cold” Barriers to T cell infiltration Tumor defence	TGF-ß1, IL10, IDO Versican Signaling CTL attack	2006 2013 2015 2022 2022	Induction of Treg Ovarian cancer invasion Structure and cells Mol mechanisms Tumor defense mechanisms	[51] [39] [31] [40] [41]
3.4. MDSC	IRF8, COX2, STAT3	2019 2021	Immunosuppression	[42] [43]
3.5. Pre-metastatic niche	VEGF, GM-CSF, exosomes (TGF-ß), TIMP1	2022 2021	Stromal reprogramming	[44] [45]
3.6. M2 TAM	IL10, TGF-ß1, MIF, PD-L1, ncRNAs	2022 2022	Inhibition of Th1, FGFs Exosomes	[46] [47]
3.7. Tolerogenic DC	Th17 responses DC	2020 2020	Pancreatic cancer Role in immunotherapy	[50] [49]
3.8. Anergic T cells	Low TCR, perforin, Fas-L, PD1	2020 2015	Checkpoint inhibition of CTL TILs, negative receptor signaling	[55] [31]
T reg cells	Secreting TGF-ß and IL-10	2022	Promotion of T cell dysfunction	[58]
3.9. Dysfunctional NK cells	Exosomes from PMN-MDSC TGF-ß and CIS	2021 2022 2022	Suppression of NK cell function in the TME Inhibition of IL-15 signaling	[59 [60] [61]
3.10. Tumor-derived factors	TGF-ß, chemokines, prostaglandins, lactic acid	2006	Recruitment of immune inhibitory cells	[62]
3.11. Metabolic barrier, hypoxia, T cell exhaustion	PD1 Dysfunctional mitochondria VEGF, bFGF HIF	2020 1971 2019	Depletion of nutrients, Accumulation of waste products Angiogenesis Role of HIF genes	[63] [32] [64]
3.12. EMT	TGF-ß1	2014	Retinopathy	[72]
3.13. Therapy resistance	Genome instability, Tumor promoting inflammation	2000 2021 2022	Hallmarks of cancer Hallmarks of cancer Hallmarks of cancer	[73] [74] [75]

bFGF = basic fibroblast growth factor; CAF = Cancer associated fibroblast; COX2 = cyclooxygenase 2; CTL = Cytotoxic T lymphocyte; ECM = Extracellular matrix; EMT = Epithelial-Mesenchymal-Transition; IDO = indoleamine-2,3-dioxygenase; IRF8 = Interferon response factor 8; MIF = Macrophage inhibition factor; MSC = Mesenchymal stem cell; ncRNA = non-coding RNA; PAI-1 = Plasminogen activator inhibitor 1; PD-L1 = Programmed cell death ligand 1; SDF-1/CXCR4 = Stroma-derived factor 1 interacting with the chemokine receptor CXCR4; STAT3 = signal transducer and activator of transcription 3; TCR = antigen-specific T cell receptor; VEGF = Vascular endothelial growth factor; TGF-ß1 = isoform 1 of Tumor growth factor ß; CIS = IL-15 signaling inhibitor; HIF = hypoxia inducing transcription factor; TIMP1 = tissue inhibitor of metalloproteinases.

**Table 3:** Counteracting immunosuppression via oncolytic NDV or cellular immunotherapy.

Feature	Mol Determinant	Year	Comment	References
4.1. Counteracting CAF	FGF2 RIG-I, IRF3/7, IFN-ß	2015 2009	Reduced RIG-I expression, Promotion of OV activity NDV susceptibility linked to reduced expression	[76] [77]
4.2. Turning cold Into hot tumors	Retinoic acid NDV induced ICD DC maturation + ICI OV treatment: TVEC + ICI NDV + ICI	2022 2022 2016 2017 2014	Increased T cell priming, trafficking and effectorfunction Immunotherapy response Combination cancer Immunotherapy Abscopal effect	[88] [79] [89] [78] [87]
4.3. Downregulating MDSC	pHN DNA plasmid vaccination	2011	HN as vaccine adjuvant, systemic function	[90]
4.4. Macrophage activation and polarization	NO, NFkB TRAIL upregulation	1996 2003	Macrophage cytotoxicity	[91] [92]
4.5. DC activation and polarization	24 TFs NFkB, Th1 cytokines	2010 2022	779 upregulated genes TAA cross-presentation	[94] [79]
4.6. T cell activation and polarization	Costimulation via NDV HN effect Induction of IFN-I IFN-I ATV-NDV-aHN-aCD28	2000 1993 1990 2005 2015	CD4 Th1 cell activation CD8 CTL activation Peptide-spec. CTL Counteracting Th2 Third signal Reactivation of anergic T cells in clinical study	[95] [96] [97] [98] [101]
4.7. NK cell activation	HN-NKp46 interaction TRAIL up	2009 2017	Stimuating NK cell cytotoxicity	[102] [103]
4.8. Targeting Rac1	Oncolytic NDV	2010 2022	Link to tumorigenesis	[104] [79]
4.9. Suppression of the metabolic barrier	Suppression by NDV of the glycolysis pathway, Delta-24-RGDOX plus IDO inhibitors, YST-OVH plus PD1 inhibitors	2020 2022 2022	Suppression of energy source for cell growth and proliferation, OV effect on Immune synapse, Localized delivery of PD-1 inhibitors	[110] [111] [112]
4.10. Breaking cancer therapy resistance and immune resistance	ICD, autophagic cell death, necroptosis, pyroptosis, ferroptosis T cell tolerance ICI Oncolysis Anti-viral immunity	2019 2018 2011 2013 2022 2011 2014 2018 2018	Breaking resistance to chemo/radiotherapy Hypoxia, SMI Angiogenesis Apoptosis and TRAIL Murine melanoma Bladder cancer Potentiation of efficacy	[113] [119] [115] [114] [118] [115] [87] [120] [121]
4.11. Recruitment of cancer-reactive MTCs from BM	Tumor cell vaccine ATV-NDV	2009 2014	Improvement of long-term survival of colon carcinoma in RCT study	[122] [123]
4.12. In situ vaccination	IO-VAC^R^ rNDV-GM-CSF	2017 2007 2021	Pretreatment with NDV + mEHT, OV effect, recruitment of DCs	[127] [128] [129]
4.13. Antibody modified NDV	rNDV-vL-vH rNDV-CD147	2008 2015	Anti-angiogenesis Anti-hepatoma	[130] [131]
4.14. NDV-loaded carrier cells	Activated T cells, Mesenchymal stem cells	2009 2020	Cross-infection of tumor cells, Altering the TME	[132] [133]
4.15. Active specific immunization	ATV-NDV post-operative vaccination, T-T cooperation	1986 1989	Prevention of metastatic spread, Augmented T cell response	[134] [135]
4.16. Adoptive cellular immunotherapy	BM MTCs GvL counteracting advanced cancer	2001 1995 2014	Therapy of human tumor xenografts, A role of vSAG-7 reactive vß6 T cells	[23] [28] [29]

ATV-NDV-aHNaCD28 = Autologous Tumor cell Vaccine modified by infection with NDV and by attachment of a bispecific costimulatory antibody; CAF = Cancer-associated fibroblast; CTL = Cytotoxic T lymphocyte; DC = Dendritic cell; ICD = Immunogenic cell death; IFN-I = Type I interferon; IFNAR = Interferon alpha receptor; IO-VAC^R^ = DC vaccine pulsed with NDV viral oncolysate; MTC = Memory T cell; NFkB = Nuclear factor kappa B; NO = Nitric oxide; PKR = Protein kinase dsRNA activated; Rac1 = A Rho GTPase; RCT = Randomized Controlled Trial; RIG-I = Retinoic acid induced gene I; TAA = Tumor-associated antigen; rNDV-GM-CSF = recombinant NDV with incorporated transgene coding for granulocyte-macrophage colony-stimulating factor; TME = Tumor microenvironment; TRAIL = Tumor necrosis factor-related apoptosis inducing ligand.

## Data Availability

Not applicable.
